# Compact Eucapnic Voluntary Hyperpnoea Apparatus for Exercise-Induced Respiratory Disease Detection

**DOI:** 10.3390/s17051139

**Published:** 2017-05-16

**Authors:** Lulu Wang, Ahmed Al-Jumaily

**Affiliations:** 1 School of Instrument Science and Opto-Electronics Engineering, Hefei University of Technology, Hefei 230009, China; 2 Institute of Biomedical Technologies, Auckland University of Technology, Auckland 1142, New Zealand; ahmed.aljumaily@aut.ac.nz

**Keywords:** eucapnic voluntary hyperpnoea, exercise-induced asthma, exercise-induced bronchoconstriction, bronchoprovocation technique, eucapnic voluntary hyperventilation, maximum voluntary ventilation

## Abstract

Eucapnic voluntary hyperpnoea (EVH) challenge provides objective criteria for exercise-induced asthma (EIA) or exercise-induced bronchoconstriction (EIB), and it was recommended to justify the use of inhaled β_2_-agonists by athletes for the Olympics. This paper presents the development of a compact and easy-to-use EVH apparatus for assessing EIB in human subjects. The compact apparatus has been validated on human subjects and the results have been compared to the conventional EVH system. Twenty-two swimmers, including eleven healthy subjects and eleven subjects who had been physician-diagnosed with asthma, were recruited from sport and recreation centers throughout Auckland, New Zealand. Each subject performed two EVH challenge tests using the proposed breathing apparatus and the conventional Phillips EVH apparatus on separate days, respectively. Forced expiratory volume in one second (FEV_1_) was measured before and after the challenges. A reduction in FEV_1_ of 10% or more was considered positive. Of the eleven subjects who were previously diagnosed with asthma, EIB was present in all subjects (100%) in the compact EVH group, while it was presented in ten subjects (90.91%) in the conventional EVH challenge group. Of the eleven healthy subjects, EIB was present in one subject (4.55%) in the compact EVH group, while it was not present in the conventional EVH group. Experimental results showed that the compact EVH system has potential to become an alternative tool for EIB detection.

## 1. Introduction

Exercise-induced bronchoconstriction (EIB) occurs during [1] or after vigorous physical activities [2], which has been reported commonly in elite athletes [3]. In 2002 Olympic Games athletes were required to demonstrate evidence of asthma or EIB to justify the use of β_2_-adrenoceptor agonist before an event [4]. Therefore, a reliable test method with an apparatus is required to accurately identify EIB in elite athletes. Several test methods have been studied to assist the diagnosis of asthma or EIB. Pharmacologic challenge has been shown to be suboptimal for identifying EIB [5]. Exercise challenge shows a low sensitivity in identifying EIB when performed in the laboratory in elite athletes [6]. Sport-specific exercise challenge is limited with respect to the standardization of the workload and the environmental conditions [7]. EVH challenge with dry air (containing 5% CO_2_, 21% O_2_, and 74% N_2_) [8] was recommended by the International Olympic Committee (IOC) as the “gold standard” for identifying EIB in elite athletes [9,10].

EVH is a challenge based on the fact that the increased ventilation rate causes bronchoconstriction in susceptible individuals [11]. This challenge was originally developed by members of the US army to test army recruits for EIB [12] and now it has been used for the diagnosis of EIB in athletes with respiratory symptoms. The EVH challenge test requires the subject to breathe rapidly (31–45 breaths per min) a quantitative amount of compressed dry air that is stored in a compressed gas cylinder at room temperature or low temperature (−20 °C to −10 °C), which desiccates the airways, mimicking the osmotic priming stimulus to EIB [13]. A pulmonary function test (measures forced expiratory volume in one second, FEV_1_) is carried out before and after the EVH test, and the subject is considered positive for EIB if FEV_1_ dropped 10% or more after the EVH test [12]. It has been recognized that hyperpnoea of dry air provides a provocative stimulus to the airway [14]. Prior studies demonstrated that cold air hyperventilation challenge is useful for subjects who complain specifically of symptoms while inspiring cold air, such as skaters and skiers [15]. However, cold air EVH challenge has low sensitivity for identifying asthmatics [16]. Phillips et al. [17] demonstrated that the temperature of the air was less important and that eucapnic balance could be maintained by admixing approximately 5% CO_2_ in the inspirited air. No significant differences were reported between cold and dry air challenges [18].

**Figure 1:**
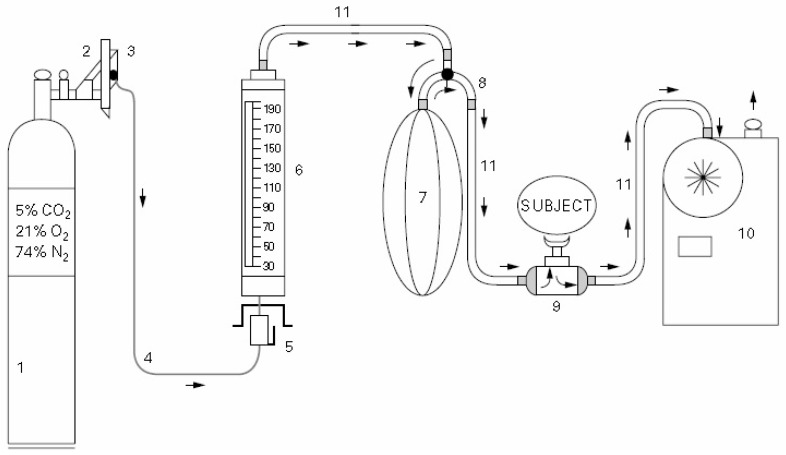
Phillips EVH challenge apparatus: 1. compressed gas mixture; 2. regulator; 3. demand resuscitator, 30–150 L/min; 4. High-pressure tubing; 5. demand valve; 6. rotameter, 30 to >200 L/min; 7. meteorological balloon; 8. metal connector with tap that allows gas to simultaneously enter and leave the balloon; 9. low resistance, low dead space volume; 10. gas meter accurate to 1 L or any other device; 11. hoses, minimum diameter 1.25 inches. Arrows indicate the direction of the gas flow.

The comparison studies between EVH and other test methods to identify EIB have been reported [19,20,21,22,23]. Although EVH challenge has been recognized as the well-established and sensitive method for detecting EIB, it has not been widely available in hospitals due to the difficult implementation method, and bulky and expensive equipment. It is also difficult for human subjects, especially non-athletes, to keep breathing for long periods with a high ventilation flow rate. The existing EVH systems were developed for laboratory-based study, which are often made of ten or more key hardware devices (see Figure 1) [24]. The modern-day protocol for EVH testing was developed at the Walter Reed Military Hospital, which is most commonly utilized in athletes [13]. For conducting an EVH challenge test, the breathing device is required to reduce the gas pressure from very high pressure (up to 160 bar) to one ambient pressure, which allows the subject to easily and safely breathe the compressed gas. Therefore, developing a breathing apparatus that has an ability to reduce the working pressure of breathing gas from the compressed gas cylinder to one ambient pressure and to deliver the compressed gas to human subjects safely are the primary factors needed to be considered in an EVH challenge system. Various sensors have been proposed for monitoring the respiratory rate, however, these sensors are not suitable for conducting an EVH challenge test [25,26,27,28,29]. To overcome some limitations of the conventional EVH challenge system, developing a computer control, compact, easy-to-use, sensitive, and cost effective apparatus that is able to perform an EVH challenge test for detecting EIB is urgently needed.

This paper presents a compact breathing apparatus for conducting an EVH challenge test to identify EIB in adults. To further support early stop of EVH challenge, the breathing apparatus contains a digital gauge (dive computer) to monitor subjects’ expiratory flow rate before, during, and after the EVH challenge. The apparatus has been tested on human subjects and compared to the conventional EVH apparatus. Each subject performed two EVH challenge tests using the developed compact breathing apparatus and Phillips apparatus over two separate days. Pre-challenge- and post-challenge-specific airway conductance and forced expiratory volume in one second (FEV_1_) were measured for the challenge and compared.

## 2. Materials and Methods

### 2.1. Breathing Apparatus Design

**Figure 2:**
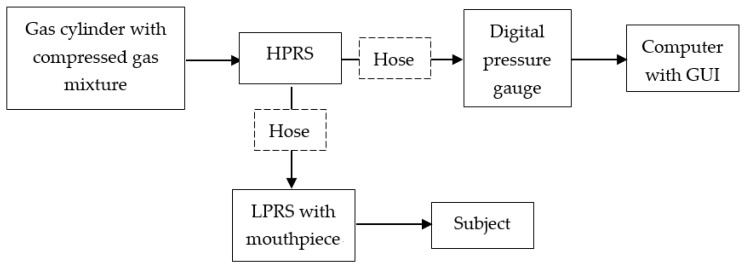
Schematic diagram of the breathing apparatus.

**Figure 3:**
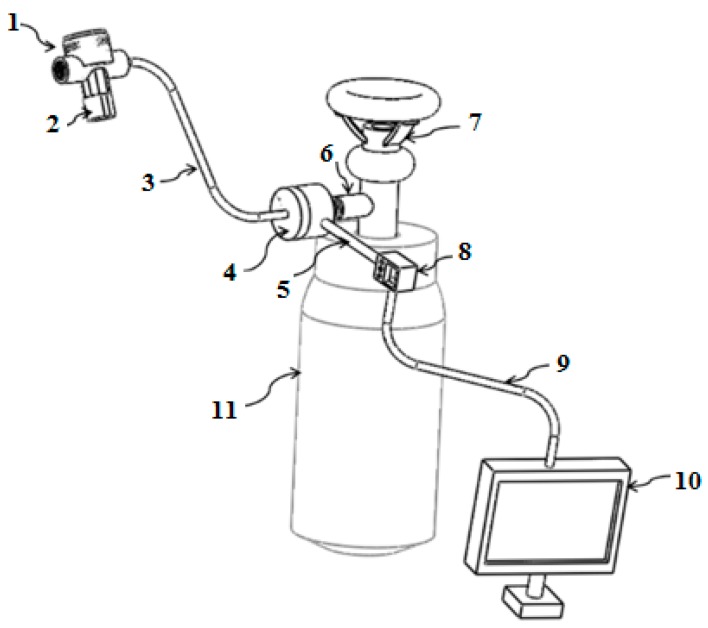
Overview of the compact breathing apparatus (spirometer is not showing): 1. LPRS; 2. mouthpiece; 3. low-pressure hose; 4. HPRS; 5. high-pressure hose; 6. connector; 7. valve; 8. dive computer (digital pressure gauge); 9. USB cable; 10: computer with GUI tool; 11. gas cylinder including compressed dry air.

Figure 2 shows the schematic diagram of the proposed breathing apparatus for the EVH challenge, which aims to reduce the high pressure (up to 160 bar) of the breathing gas stored in a gas cylinder to one ambient pressure and deliver the compressed gas to human subjects safely. Referring to Figure 3, the apparatus consists of a gas cylinder with compressed dry air, a diaphragm-based high-pressure reducing sensor (HPRS) with a high-pressure hose (minimum diameter 1.25 inches, minimum length 8 inches), a digital pressure gauge, a diaphragm-based low-pressure reducing sensor (LPRS) with low pressure hose (minimum diameter 1.25 inches, minimum length 8 inches), and a mouthpiece.

**Figure 4:**
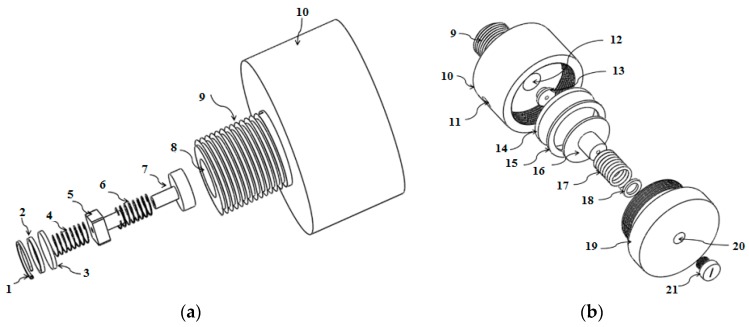
(**a**) Cross-sectional view of the high-pressure chamber of the HPRS: 1. retainer; 2. O-ring; 3. diaphragm; 4. spring; 5. high pressure filter; 6. high pressure spring; 7. high pressure seat; 8. input port; 9. cylinder fitting; 10. top body cover of HPRS; and (**b**) the cross-sectional view of the intermediate pressure chamber of the HPRS: 9. cylinder fitting; 10. top body cover of HPRS; 11. outlet port; 12. valve; 13. pin; 14. low pressure diaphragm; 15. O-ring; 16. low pressure seat; 17. low pressure spring; 18. O-ring; 19. cap; 20. outlet port; 21. pin.

**Figure 5:**
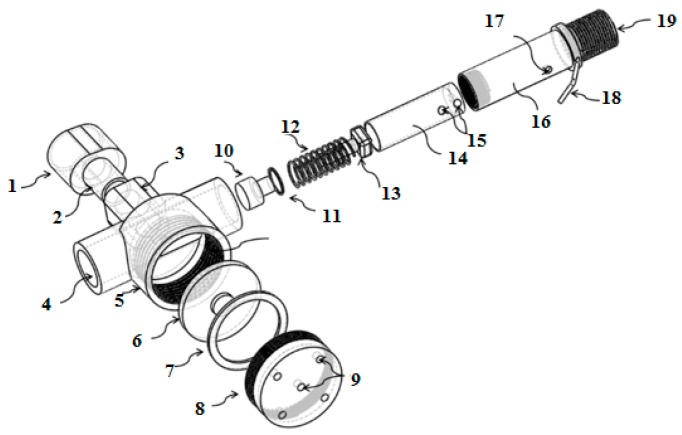
LPRS with mouthpiece: 1. mouthpiece; 2. mouthpiece fitting; 3. output port of LPRS; 4. low air input hole; 5. body cover of LPRS; 6. diaphragm; 7. seal; 8. elastic diaphragm cover; 9. exhaust ports; 10. pressure control knob; 11. O-ring; 12. spring; 13. seat; 14. pressure house; 15. air holes; 16. tubing house; 17. air hole; 18. lever; 19. inlet port.

Figure 4 and Figure 5 show the diaphragm-based HPRS and LPRS, which were made of steel and silicon. If there is no LPRS in the system, the HPRS is closed with pin 21. The HPRS was designed to reduce the gas pressure from a high pressure (approximately 140–160 bar) to a low pressure level (approximately 1–1.5 bar). The high pressure outlet port has an operating area larger than that of a low-pressure outlet port. LPRS was designed to take the compressed dry air from HPRS and further reduce it to one ambient pressure, which allows the subject to easily and safely breathe dry air. The gas from the HPRS is delivered into the tubular housing and flows through poppet valve to the subject’s mouth.

A graphical user interface (GUI) tool was developed for analyzing the challenge performance. The “target end pressure (P_TE_)” was introduced as a guideline to stop the challenge based on:

P_TE_ = P_S_ − P_cylinder_V_T_/V_cylinder_ (1)

where P_S_ means the gas cylinder pressure before the challenge, P_cylinder_ is the working pressure, and V_cylinder_ is the capacity of the gas cylinder.

The target ventilation volume (V_T_) of gas delivered to the subject can be calculated:

V_T_ = Target Flow Rate × (T_2_ − T_1_) (2)

where Target Flow Rate represents the target ventilation flow rate or flow rate, T_1_ and T_2_ are the challenge start time and finish time, respectively.

The target ventilation flow rate can be calculated as:

Target Flow Rate = Multi-factor × FEV_1_ (3)

The conventional Phillips EVH apparatus [18] involves a single level of ventilation with a target of 30 × FEV_1_. The target ventilation rate is normally equivalent to the ventilation factor × FEV_1_ value. Most elite athletes should easily achieve 25 × FEV_1_, and most asthmatics need only breathe 21 × FEV_1_ to have an abnormal response [13]. Therefore, the multi-factor for the proposed breathing apparatus can be 21–30. The target ventilation rate is equivalent to Multi-factor × FEV_1_, which is equivalent to 85% of the maximum voluntary ventilation.

The actual total volume of gas respired (V_E_) by the subject during the challenge test is based on:

V_E_ = (P_S_ − P_TE_ ) × V_cylinder_/P_cylinder_ (4)

To measure the cylinder pressure of dry air at significantly low cost, a dive computer was purchased from Sherwood Scuba (Dolphin Aquatics, Huntsville, AL, USA) to work as a digital pressure gauge. During operation, the user presses the bottom of the LPRS to release unwanted gas located in the connecting holes through exhaust port 9 (located at the bottom of the LPRS) before opening the cylinder valve. This step aims to remove “dead-space” volume. The user then opens the cylinder valve to start collecting data.

When the subject inhales, gas flows from the cylinder into the high-pressure gas chamber through a valve and then flows into the intermediate pressure chamber. The valve between these two chambers stays open until the gas in the intermediate pressure chamber reaches the required pressure at which the valve closes, preventing the high-pressure gas from the cylinder to flow into the intermediate pressure chamber.

When the subject inhales, gas from the intermediate chamber is released to the LPRS and the pressure drops, which allows the valve to open again. Gas flows from the high-pressure chamber into the intermediate-pressure chamber until the pressure in the intermediate chamber rises to the required pressure, at which time the valve closes again. The pressure inside the LPRS decreases, causing the diaphragm inside the LPRS to depress and actuate a lever. This lever opens the valve allowing gas to come into the LPRS and flow to the subject. When the subject exhales, the diaphragm is extended and the exhaled gas leaves the LPRS through exhaust port 9 located at the bottom of the LPRS.

### 2.2. Methods

#### 2.2.1. Human Subjects and Study Design 

Twenty-two adult swimmers (eleven women and eleven men) were randomly recruited from sport and recreation centers throughout Auckland, New Zealand. All subjects had a negative history for cardiovascular disease and other chronic illnesses, except eleven subjects who had been physician-diagnosed with asthma and two of them were treated with β_2_-agonists. This pilot study was approved by the Auckland University of Technology’s ethics review board (protocol number 14/47).

Each subject performed two EVH challenge tests using the proposed breathing apparatus and the conventional Phillips EVH apparatus on separate days, respectively. The EVH challenge was performed according to the American Thoracic Society (ATS) guidelines [30]. Written informed consent were obtained from all subjects. All individuals were analyzed outside the pollen season and with no air pollution exposure.

#### 2.2.2. Pre-Challenge

Before the study, each subject completed a demographic questionnaire on the history of past and current respiratory symptoms based on the European Community Respiratory Health Survey Questionnaire [31], all subjects were weighed, and then the body mass index (BMI, kg/m^2^) was calculated. The use of inhaled corticosteroids, β_2_-agonists, and leukotriene receptor antagonists during the last three months was recorded. The subjects were instructed on how to prepare for the two EVH challenge tests, they were also instructed to cease short-acting β_2_-agonists 8 h before the test, long-acting β_2_-agonists 24 h before the test, and leukotriene receptor antagonists 72 h before the test [1]. They were required not to use inhaled steroids on the day of the test and to avoid vigorous exercise, heavy meals, caffeine containing food or beverages and nicotine for 4 h before the test [32].

Pulmonary function test was performed according to ATS/European Respiratory Society (ERS) guidelines to measure FEV_1_ (pre-challenge FEV_1_). The pre-challenge FEV_1_ was measured three times before the EVH challenge with the subjects seated in a chair directly next to the treadmill. A spirometer (ndd Medical Technologies, Andover, MA, USA) was used to collect all spirometry measurements. Maximal flow volume loops were recorded before and at 5, 10, and 15 min after the EVH challenge according to international guidelines for spirometry [33].

Three acceptable FEV_1_ values (which should agree within 3% for adults) were performed 30 min prior to each challenge, and the highest value was used to calculate the airway response. The test involves taking deep breaths and blowing all of the subject’s air out into a spirometer that measures subjects’ lung capacity and air flow rate. The total lung volume was measured by having the subject perform brief panting maneuvers into a mouthpiece. Subjects were encouraged to hold their breath for 10 s before the pulmonary function test.

#### 2.2.3. Conventional EVH Challenge

The conventional EVH challenge was performed using the Phillips apparatus [18]. This protocol involves a single level of ventilation with a target of 30 × FEV_1_. The subjects were required to hyperventilate dry air at room temperature, at 85% maximal voluntary ventilation (MVV) for a total duration of 6 min. FEV_1_ was measured 5, 10, and 15 min after the test. The conventional EVH challenge apparatus components were purchased from local regulator suppliers (Air Liquide, Auckland, New Zealand) and assembled at the mechanical workshop, Auckland University of Technology, Auckland, New Zealand.

#### 2.2.4. Compact EVH Challenge

A Sherwood scuba diving computer (Dolphin Aquatics, Huntsville, AL, USA) was purchased to work as the digital pressure gauge, a GUI tool was developed using MATLAB software (Natick, MA, USA), and the experimental prototype was fabricated and assembled at the mechanical workshop, Auckland University of Technology, New Zealand. The compressed breathing cylinder with dry air was purchased from the local gas supplier in Auckland.

The compact EVH challenge test was performed using the proposed breathing apparatus, and the GUI tool was employed to assess and analyze the challenge results. Each subject was seated comfortably, and FEV_1_ was measured 5, 10, and 15 min after the test. The target ventilation was calculated on the basis of the measured FEV_1_. Prior to the challenge, the user recorded the setup information to calculate the start pressure, target expiratory flow rate, target ventilation flow rate, target ventilation, and target end pressure by using the GUI. Using the breathing sensor, the subjects inhaled dry air at room temperature until the pressure of the gas stored in the cylinder reached the “target end pressure”.

### 2.3. Measurement of the Airway Response

The FEV_1_ was measured before, and in duplicate at 5, 10, and 15 min after, each challenge. The percentage changes in FEV_1_ were calculated for each challenge by taking the lowest measurement recorded at 5, 10, and 15 min after each challenge. The percentage fall in FEV_1_ (%fall in FEV_1_) is:

(5) $${\%\mathrm{fall}\;\mathrm{in}\;\mathrm{FEV}}_{1}=\frac{{\mathrm{FEV}}_{1b}-{\mathrm{FEV}}_{1a}}{{\mathrm{FEV}}_{1b}}\times 100$$

where ${\mathrm{FEV}}_{1b}$ is FEV_1_ before the challenge and ${\mathrm{FEV}}_{1a}$ is the lowest value for FEV_1_ measured in the 15 min following the challenge. A fall in FEV_1_ of 10% or more defines a diagnosis of EIB [12], and a fall in FEV_1_ of 15% or more defines a diagnosis of EIA [9].

## 3. Results

Twenty-two subjects (see Table 1), including eleven subjects who were previously diagnosed with asthma, were recruited and completed two EVH challenge tests using the developed breathing apparatus and Phillips apparatus on two separate days. Five subjects complained of intermittent respiratory symptoms, such as dyspnea, wheezing, and coughing. Two subjects were on medication (β_2_-agonists) during the last three months before the study. They were instructed to stop medication 24 h before the test. No subject complained of discomfort owing to the breathing apparatus, only citing a “dry throat” arising from the fact they had inhaled compressed dry air.

**Figure 6:**
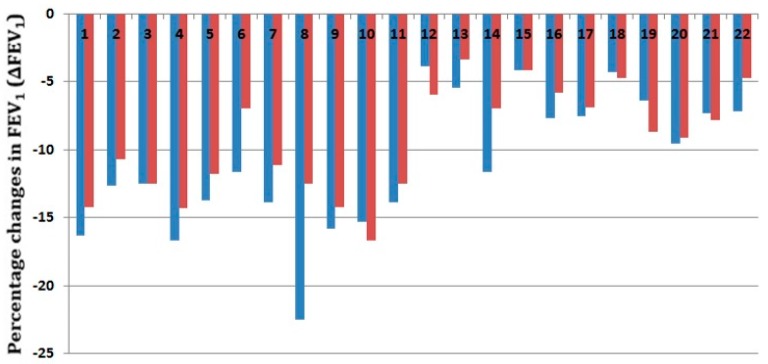
Percentage changes of FEV_1_ after compact EVH and conventional EVH challenge tests, blue bar means compact EVH challenge test, red bar means conventional EVH challenge test.

Of the eleven subjects who were previously diagnosed with asthma, EIB was present in all subjects (100%) and EIA was presented in five subjects (22.73%) in the compact EVH group, while EIB was present in ten subjects (90.91%) and EIA was presented in one subject (4.55%) in the conventional EVH challenge group (see Figure 6). Of the eleven health subjects, EIB was present in one subject (4.55%) in the compact EVH challenge group, while it was not present in the conventional EVH group, and no EIA was presented in both groups (see Table 2).

**Figure 7:**
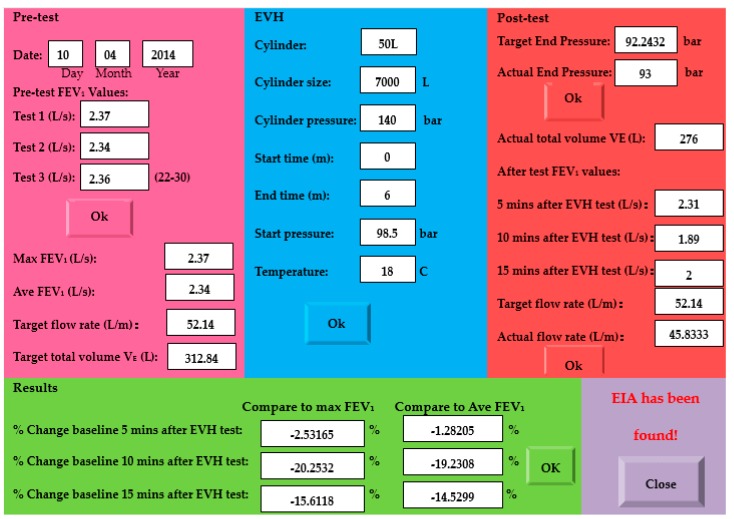
EVH challenge result.

Figure 7 shows the compact EVH challenge result of a 32-year-old female participant who was previously diagnosed with asthma, but without medications for at least six months before the study. The start test pressure was 98.5 bar. According to previous studies [13], she was given a compact EVH challenge with a target expiratory flow rate of 22 times her maxima FEV_l_, the target ventilation for 6 min was 312.84 L, and the target end pressure was 92.2432 bar. The participant stopped the challenge when the cylinder pressure reached 93 bar. She had three pulmonary functions at 5, 10, and 15 min after the EVH test, respectively, and the lowest FEV_l_ at different times was recorded. The female participant had a 20.25% and 15.61% decrease in her FEV_l_ from baseline at 10 and 15 min after the challenge, respectively. Her actual expiratory flow rate (45.833 L/m) and actual total volume of gas respired (275 L) for 6 min are smaller than the target values. She was informed that she would be at high risk of EIA.

## 4. Discussion

This study investigated a compact EVH challenge apparatus to identify EIB. The conventional EVH challenge system uses a “time control” implementation method that requires the subjects to rapidly breathe dry air for 6–8 min. However, most asthmatics need less dry air than normal people or athletes. It is also difficult for human subjects, especially non-athletes, to keep breathing for a long period with a high ventilation flow rate. The existing “time control” ventilation system requires all measurements to be made accurately in real-time and displayed so that participants can receive feedback on their target minute ventilation. Thus, minute ventilation must be monitored.

Different from the apparatus presented in [34], the proposed apparatus uses a compact EVH challenge method and performs an EVH challenge using a GUI tool. The expensive data-acquisition unit and digital pressure gauge are replaced by the GUI tool and dive computer in the compact EVH system. The dive computer monitors the ventilation rate and cylinder pressure in real-time and stops the challenge immediately if the substantial ventilation rate is increased; thus, additional monitoring equipment is not required to maintain CO_2_ and O_2_ levels.

Compared to conventional EVH system, the proposed apparatus does not require high-cost components, as the expensive compressed gas regulator, data acquisition unit, and gas meter were replaced by HPRS, LPRS, a dive computer, and a GUI tool, which have significantly reduced the system running cost. The GUI produces a report of all of the test parameters and patient performance, and allows for an auto-generated interpretation of results. The GUI tool can generate the actual total volume of gas respired by the subjects in real-time. The actual ventilation volume is normally smaller than the target ventilation volume which is not predicted by existing EVH; therefore, patients who are at high risk of EIB may not be identified if they did not inhale enough dry air within 6 min. The GUI tool monitors all necessary information. Such a design provides a much safer testing environment. Some significant advantages of the design include compactness, cost-effectiveness, safety, and ease of use and manufacture.

The major limitation of the compact EVH system is that the digital dive computer (cost-effective digital gauge) cannot stop working immediately (it has to run for 2 h) after the EVH challenge. Another limitation of this study is that there was only a small number of people involved in the pilot study, which may potentially affect the result. Thus, the difference found in athletes may be somewhat different in situations that affect these factors (e.g., obesity, lung disease, and hypothyroidism). These situations, and the relationship between the two tests in such conditions, require further investigation.

Previous studies showed that a multi-factor of 21–30 is a suitable range to bronchoprovocation EIB in EVH, and most elite athletes should easily achieve 25 × FEV_1_, while most asthmatics need only breathe 21 × FEV_1_ to have an abnormal response [13]. Finding the best multi-factor of the EVH challenge test is related to the clinical study of the EVH challenge across a large number of people; the best multi-factor may be related to sex, age, and other health conditions. This project did not focus on clinical studies and only a small number of people were involved. According to previously-published results [13], the authors used a multi-factor of 22 for participants who had been physician-diagnosed with asthma (such as the result shown in Figure 7) and a multi-factor of 25 for healthy subjects to ensure they would get enough dry air to bronchoprovocation EIB in EVH.

The percentage fall in FEV_1_ shows the sensitivity of the apparatus and severity of EIB. The proposed apparatus seems has an ability to produce a higher percentage fall in FEV_1_ than the conventional EVH apparatus, especially in subjects who had been physician-diagnosed with asthma. Results presented in Subject 8 showed that the compact EVH apparatus provides a much higher percentage fall in FEV_1_ compared to the conventional EVH apparatus, which may be caused by the measurement error. However, it is difficult to conclude that the compact EVH apparatus is more sensitive than the conventional EVH for identifying EIB in adult athletes due to a small number of subjects that were involved in this pilot study. The authors would like to indicate that this project did not focus on clinical trial studies. Their intention was to test whether the proposed compact breathing apparatus can be employed in an EVH challenge test to identify EIB or EIA in adult subjects. Importantly, experimental results confirmed that the proposed breathing apparatus is compact, safe, and convenient to use without side effects to subjects, which has the potential to become an alternative testing tool for screening EIB. Clinical trials of exercise-induced respiratory diseases using the developed EVH apparatus across a large number of people is planned in the future.

## 5. Conclusions

A new compact breathing apparatus for conducting an EVH challenge test to identify EIB was presented in this paper. The compact EVH apparatus uses a pressure control method to stop the challenge, and it is compact, cost-effective, safe, and convenient to use without side effects to subjects. No subject complained of discomfort owing to the proposed breathing apparatus, only citing a “dry throat” arising from the fact they had inhaled compressed dry air. Experimental results showed that the compact breathing apparatus can be used to perform an EVH challenge test in adults which may have the potential to become a useful tool to assess EIB across a larger population.

## Figures and Tables

**Table 1:** Demographic characteristics of subjects.

	All Participants	Male Participants	Female Participants
Number (n)	22	11	11
Age (years)	27.3 ± 3.0	28.2 ± 2.54	26.3 ± 3.3
Body Mass Index (kg/m^2^)	20.4 ± 1.60	20.7 ± 0.88	20.9 ± 2.09
Smoking (yes)	2 (9.09%)	2 (18.2%)	0 (0%)
Shortness of breath	3 (13.64%)	1 (9.09%)	2 (18.2%)
Wheezing	5 (22.73%)	2 (18.2%)	3 (13.64%)
Cough	3 (13.64%)	2 (18.2%)	3 (13.64%)
Night symptoms	3 (13.64%)	2 (18.2%)	1 (9.09%)

**Table 2:** Experimental results.

Subject	Baseline FEV_1_ (Liters)	Predicted FEV_1_	Compact EVH ΔFEV_1_ (%)	Conventional EVH ΔFEV_1_ (%)
1	4.78	103	−16.32	−14.23
2	5.15	120	−12.62	−10.68
3	4.8	104	−12.5	−12.5
4	4.2	97	−16.67	−14.29
5	5.1	120	−13.73	−11.76
6 *	4.3	100	−11.63	−6.98
7	3.6	79	−13.89	−11.11
8	4	105	−22.5	−12.5
9	3.8	86	−15.79	−14.21
10	3.78	85	−15.34	−16.67
11	3.6	78	−13.89	−12.5
12	4.89	105	−3.89	−5.93
13 *	4.76	103	−5.46	−3.36
14	4.3	100	−11.63	−6.98
15	4.8	104	−4.17	−4.17
16	5.2	125	−7.69	−5.77
17	5.2	116	−7.5	−6.9
18	4.8	97	−4.3	−4.7
19	4.38	99	−6.39	−8.68
20	4.18	97	−9.57	−9.09
21	4.1	98	−7.32	−7.8
22	4.2	101	−7.14	−4.76

* Subject who had medication (β_2_-agonists) in the last one year before the study; Compact EVH ΔFEV_1_ (%): percentage changes of FEV_1_ after compact EVH challenge; Conventional EVH ΔFEV_1_ (%): percentage changes of FEV_1_ after conventional EVH challenge.
